# Homophobia: Is it a heterosexual “privilege”?

**DOI:** 10.1192/j.eurpsy.2021.1457

**Published:** 2021-08-13

**Authors:** I. Kourtesis

**Affiliations:** Department Of Adult Psychiatry, Psychiatric Hospital of Athens “Dafni”, ATHENS, Greece

**Keywords:** LGBT, stigmatization, discrimination, internalized homophobia

## Abstract

**Introduction:**

The context of stigma, in which many homosexuals live, exposes them to discrimination/stigmatization and promotes the internalization of negative attitudes about sexuality. Battle feelings of rejection and isolation may lead to the development of internalized homophobia (IH).

**Objectives:**

To elucidate the risk factors of IH and how the latter affects mental health.

**Methods:**

Literature review (PubMed).

**Results:**

IH has been associated with lower levels of emotional stability, rejection sensitivity, impairments in emotion regulation and a tendency to turn against the self. Studies have connected IH to depression, poor self/relationship wellbeing, sexual discrimination, addictions, shame, body dissatisfaction, suicidal ideation, binge eating/drinking, partner violence and victimization. Higher levels of attachment anxiety and avoidance, outness, religiosity and internalized stigma were correlated with higher IH levels. The key factors relating to suicide include lack of acceptance by family and/or self, negative feelings about sexuality/gender and appearance dissatisfaction. In traditional/religious societies, heterosexual orientation is a strong norm and homosexuality is considered unacceptable leading LGBT individuals to report higher depressive symptoms and increased levels of IH. Due to heterosexist ideals, IH is a predictor for heterosexual marital intention, protests against LGBT and use of masculinity as a compensatory strategy.

**Conclusions:**

Policies in support of individuals who have recently come out should be improved to reduce the development/effects of IH and take the social and sexual environments of rural gay men into account considering ways to increase service accessibility (e.g internet). Future research is needed to further understand the association between IH and mental health, social and cognitive mechanisms.

